# Comparison of angiopoietin-like protein 3 and 4 reveals structural and mechanistic similarities

**DOI:** 10.1016/j.jbc.2021.100312

**Published:** 2021-01-20

**Authors:** Kathryn H. Gunn, Aspen R. Gutgsell, Yongmei Xu, Caitlin V. Johnson, Jian Liu, Saskia B. Neher

**Affiliations:** 1 Department of Biochemistry and Biophysics, University of North Carolina, Chapel Hill, North Carolina, USA; 2 Division of Chemical Biology and Medicinal Chemistry, Eshelman School of Pharmacy, University of North Carolina, Chapel Hill, North Carolina, USA; 3 Department of Chemistry, University of North Carolina, Chapel Hill, North Carolina, USA

**Keywords:** enzyme kinetics, small-angle X-ray scattering (SAXS), lipoprotein metabolism, heparin-binding protein, protein purification, lipoprotein lipase (LPL), very-low-density lipoprotein (VLDL), noncompetitive inhibition, CAD, coronary artery disease, DGGR, 1,2-Di-O-lauryl-*rac*-glycero-3-glutaric acid 6′-methylresorufin ester, LPL, lipoprotein lipase, NEFA, nonesterified fatty acid, SAXS, small-angle X-ray scattering, SEC, size-exclusion chromatography, VLDL, very-low-density lipoprotein

## Abstract

Elevated plasma triglycerides are a risk factor for coronary artery disease, which is the leading cause of death worldwide. Lipoprotein lipase (LPL) reduces triglycerides in the blood by hydrolyzing them from triglyceride-rich lipoproteins to release free fatty acids. LPL activity is regulated in a nutritionally responsive manner by macromolecular inhibitors including angiopoietin-like proteins 3 and 4 (ANGPTL3 and ANGPTL4). However, the mechanism by which ANGPTL3 inhibits LPL is unclear, in part due to challenges in obtaining pure protein for study. We used a new purification protocol for the N-terminal domain of ANGPTL3, removing a DNA contaminant, and found DNA-free ANGPTL3 showed enhanced inhibition of LPL. Structural analysis showed that ANGPTL3 formed elongated, flexible trimers and hexamers that did not interconvert. ANGPTL4 formed only elongated flexible trimers. We compared the inhibition of ANGPTL3 and ANGPTL4 using human very-low-density lipoproteins as a substrate and found both were noncompetitive inhibitors. The inhibition constants for the trimeric ANGPTL3 (7.5 ± 0.7 nM) and ANGPTL4 (3.6 ± 1.0 nM) were only 2-fold different. Heparin has previously been reported to interfere with ANGPTL3 binding to LPL, so we questioned if the negatively charged heparin was acting in a similar fashion to the DNA contaminant. We found that ANGPTL3 inhibition is abolished by binding to low-molecular-weight heparin, whereas ANGPTL4 inhibition is not. Our data show new similarities and differences in how ANGPTL3 and ANGPTL4 regulate LPL and opens new avenues of investigating the effect of heparin on LPL inhibition by ANGPTL3.

Lipoprotein lipase (LPL) plays a central role in the regulation of whole-body energy balance by hydrolyzing triglycerides from circulating lipoproteins to provide free fatty acids to tissues. LPL activity is differentially regulated in adipose *versus* muscle tissue depending on nutritional state in order to ensure appropriate distribution of available energy throughout the body (1). Specifically, LPL activity is downregulated in adipose tissue during fasting to direct triglyceride-rich lipoproteins to muscle tissue for fatty acid oxidation (2, 3). Conversely, in the postprandial state, LPL activity increases in adipose tissue but decreases in muscle tissue. This allows utilization of glucose by muscle tissue and fat storage in adipose tissue (3). This tissue-specific regulation is facilitated by three members of the angiopoietin-like protein (ANGPTL) family of proteins: ANGPTL3, ANGPTL4, and ANGPTL8 (4). Each protein is an inhibitor of LPL, and each has a different pattern of expression. ANGPTL3 is highly expressed in the liver and is largely insensitive to nutritional state (5). Upon fasting, ANGPTL4 is upregulated in the adipose tissue (6). ANGPTL8 is upregulated postprandially and expressed in the liver and adipose (7); it must interact with ANGPTL3 to inhibit LPL (8, 9, 10). Recent work has shown that ANGPTL8 can also form a complex with ANGPTL4 that prevents LPL inhibition (11).

Genome-wide association studies in humans have identified robust links between lipid profiles and variants in ANGPTL3, ANGPTL4, and ANGPTL8. Mutations resulting in the loss of ANGPTL3 function are associated with lower plasma triglycerides and protection from coronary artery disease (CAD) (12, 13, 14). Similarly, individuals with inactivating mutations in ANGPTL4 have lower triglycerides and a lower incidence of CAD than noncarriers (15, 16). Coding variants in ANGPTL8 are very rare, and were not associated with CAD risk, but were associated with high-density lipoprotein–cholesterol and triglyceride levels (17). However, high circulating levels of ANGPTL8 were found to be associated with a lower risk of cardiovascular events (18).

ANGPTL3 and ANGPTL4 have similarities that extend beyond their effects on lipid profiles in humans. They are both secreted proteins with many similar structural characteristics and share 29% sequence identity. Both have an N-terminal coiled-coil domain and a C-terminal fibrinogen-like domain separated by a furin cleavage site (19, 20). The N-terminal coiled-coil domain of both proteins inhibit LPL *in vitro* (21, 22). Within this N-terminal domain, both ANGPTL3 and ANGPTL4 share a conserved LPL inhibition motif (23). With so many similarities, some puzzling differences emerge between ANGPTL3 and ANGPTL4. One unanswered question is why the two inhibitors show such different potencies *in vitro*. Another unanswered question surrounds the structure and oligomeric form of the N-terminal domains of both inhibitors, which are not known.

In previous work, comparisons of *in vitro* LPL inhibition by ANGPTL3 and ANGPTL4 showed that ANGPTL4 inhibition of LPL was up to 100× more potent than ANGPTL3 (11, 24, 25, 26). However, in humans, a loss-of-function allele in ANGPTL3 resulted in a greater decrease in plasma triglyceride levels and a greater decrease in CAD risk relative to carriers of a loss of function allele of ANGPTL4 (12, 15). Differences have also been observed in the abilities of ANGPTL3 and ANGPTL4 to inhibit LPL in the presence of other proteins and additives. For example, heparin was found to protect LPL from inhibition by ANGPTL3 but not from inhibition by ANGPTL4 (24, 25). It was proposed that this protection was due to competitive binding with heparin to LPL, resulting in occlusion of the ANGPTL3 binding site on LPL (24). Glycosylphosphatidylinositol anchored high-density lipoprotein binding protein 1 (GPIHBP1), which binds LPL and anchors it to the capillary, has also been found to decrease the ability of ANGPTL3 and ANGPTL4 to inhibit LPL (27).

The mechanism of ANGPTL3 and ANGPTL4 inhibition of LPL has remained unclear, partially due to a variety of methods for assessing their inhibitory activity. Different studies have arrived at conflicting conclusions about whether both ANGPTL3 and ANGPTL4 are irreversible or reversible LPL inhibitors (24, 28, 29, 30). When tested on a nonphysiological fluorescent substrate, ANGPTL4 was found to be a noncompetitive inhibitor (29) and ANGPTL3 was found to be a reversible inhibitor (24). By contrast, both ANGPTL3 and ANGPTL4 were suggested to irreversibly unfold LPL by hydrogen–deuterium exchange mass spectrometry (28, 30). It is clear that more data are needed to elucidate how ANGPTL3 and ANGPTL4 inhibit LPL on natural substrates.

Crystal structures of the C-terminal fibrinogen like domains of both ANGPTL3 and ANGPTL4 have been resolved, but little is known about the structure and oligomeric states of the N-terminal domains of both proteins (31). One study showed that the N-terminal domains of both ANGPTL3 and ANGPTL4 elute from size-exclusion chromatography (SEC) in a broad peak of high-molecular-weight oligomers (32). In a later study, SEC suggested that the N-terminal domain of ANGPTL4 forms hexamers (24). By contrast, a study that compared the apparent molecular weight of wildtype, mammalian-produced N-terminal domain ANGPTL4 using reducing and nonreducing SDS-PAGE found that ANGPTL4 formed disulfide-bonded dimers and tetramers (20).

In this work, we undertook a biochemical and biophysical study of the N-terminal domains of ANGPTL3 and ANGPTL4 to address these outstanding questions. We found that ANGPTL3 associates with a DNA contaminant and removal of this DNA contaminant enhances ANGPTL3 inhibition of LPL. We found that ANGPTL3 adopts two distinct oligomeric states, hexamer and trimer, whereas ANGPTL4 was only observed as a trimer by SEC–multiangle light scattering (MALS). Using small-angle X-ray scattering (SAXS) we found both ANGPTL3 and ANGPTL4 adopt elongated and flexible structures in solution. We assessed the mechanism of LPL inhibition by both ANGPTL3 and ANGPTL4 on human very-low-density lipoprotein (VLDL) using an enzyme-coupled reaction to report on real-time liberation of free fatty acids. This revealed both ANGPTL3 and ANGPTL4 are noncompetitive inhibitors. We also investigated the different effects heparin has on ANGPTL3 and ANGPTL4 inhibition. These data provide new insight into how these two inhibitors influence LPL activity.

## Results

### DNA blocks ANGPTL3 inhibition of LPL

**Figure 1:**
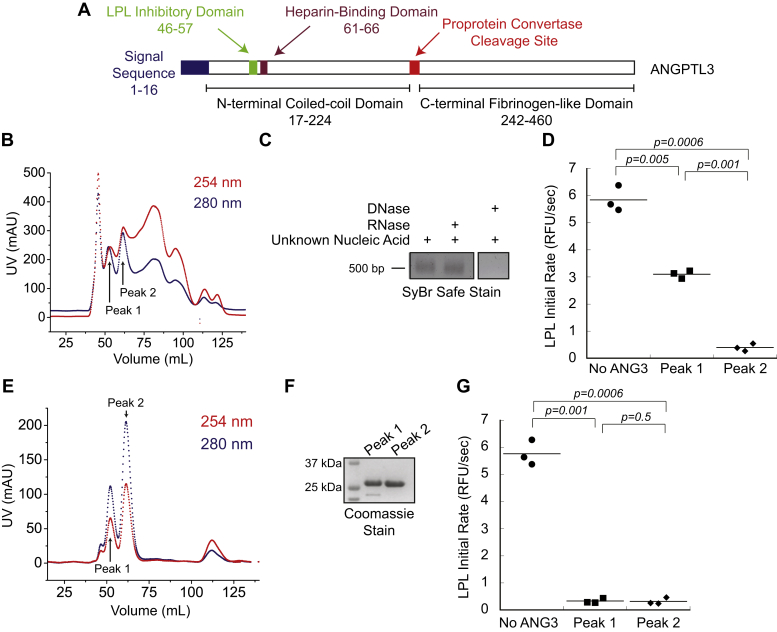
**DNA contamination reduces ANGPTL3 inhibition potency.***A*, schematic of ANGPTL3, highlighting the N-terminal construct used for this work. *B*, representative ANGPTL3 SEC elution profile following nickel affinity purification. ANGPTL3 protein (280 nm, *blue*) eluted as two peaks, labeled peak 1 and peak 2. The ANGPTL3 was contaminated with nucleic acid (254 nm, *red*). *C*, the ANGPTL3 nucleic acid contaminant from ANGPTL3 peak 1 was visualized on an agarose gel. The contaminant was sensitive to DNase, but not RNase, indicating the contaminant was DNA. *D*, lipoprotein lipase inhibition with DNA-contaminated ANGPTL3 peak 1 and peak 2 significantly differ. *E*, an additional MonoQ anion-exchange purification step was performed between the nickel affinity column and SEC. A representative SEC trace shows that the ANGPTL3 DNA contamination was substantially reduced (254 nm, *red*), allowing better resolution of the ANGPTL3 peak 1 and peak 2 proteins (280 nm, *blue*). *F*, ANGPTL3 peak 1 and peak 2 were visualized using SDS-PAGE, showing both peak 1 and peak 2 contain ANGPTL3 (26.8 kDa). *G*, after removal of DNA, both ANGPTL3 peak 1 and peak 2 inhibit lipoprotein lipase equivalently. SEC, size-exclusion chromatography.

In previous *in vitro* studies, ANGPTL3 was shown to be a less potent inhibitor of LPL than ANGPTL4 (24, 25, 26). Most of these studies used the N-terminal domain of ANGPTL3 (∼26 kDa) recombinantly produced in *Escherichia coli* and purified using nickel chromatography (24, 25). We repeated this protocol and included a SEC step for additional purification (24). Unexpectedly, we found that N-terminal ANGPTL3 (Fig. 1*A*) copurified with a significant nucleic acid contamination, as observed by absorbance at 254 nm (Fig. 1*B*). The contaminating nucleic acid was sensitive to DNase treatment, but resistant to RNase treatment, indicating that ANGPTL3 was bound to DNA (Fig. 1*C*). We also observed two peaks in the included volume of the SEC that contained ANGPTL3 (Fig. 1*B*). We pooled and concentrated the ANGPTL3 from each peak separately and assessed *in vitro* LPL inhibition using human VLDL as a substrate. These experiments showed that ANGPTL3 peak 1 had a significantly lower LPL inhibition activity than peak 2 (Fig. 1*D*). In order to remove the contaminating DNA from the ANGPTL3 preparation, we added an additional purification step, MonoQ anion-exchange chromatography, which led to a significant reduction in DNA and cleaner ANGPTL3 as observed from the 254 nm trace from the subsequent SEC (Fig. 1*E*). Of interest, DNA-free ANGPTL3 still eluted in two distinct peaks from the SEC after this additional anion-exchange step. Both peak 1 and peak 2 contained >95% pure ANGPTL3 (Fig. 1*F*). Next, ANGPTL3 from peaks 1 and 2 were tested for inhibition of LPL acting on VLDL. These assays showed that protein from both peaks were now equally as effective at inhibiting LPL (Fig. 1*G*). Thus, removal of DNA from ANGPTL3 increased its potency as an LPL inhibitor.

### ANGPTL3 forms trimers and hexamers and ANGPTL4 forms trimers

**Figure 2:**
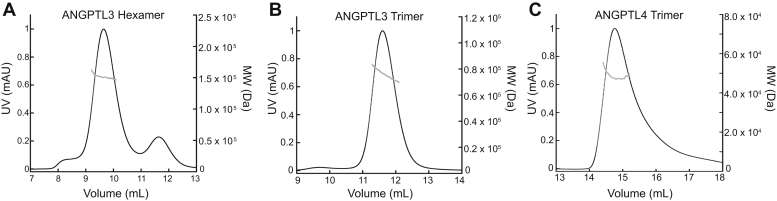
**ANGPTL3 forms noninterconvertible hexamers and trimers and ANGPTL4 forms a trimer.** SEC purified (*A*) ANGPTL3 peak 1 and (*B*) ANGPTL3 peak 2 were separately concentrated and reinjected for analysis with SEC–multiangle light scattering. Peak 1 had a molecular weight (MW) of 151 kDa, indicating a hexamer of ANGPTL3 (monomer 26.8 kDa). Peak 2 had a MW of 76 kDa, indicating a trimer of ANGPTL3. The ANGPTL3 trimers and hexamers did not interconvert when reinjected on SEC. Owing to the elution being close together, a small amount of trimer and hexamer can be seen after the peaks were separated and reinjected. *C*, SEC purified ANGPTL4 was concentrated and injected for SEC–multiangle light scattering analysis. ANGPTL4 resolved as a single peak with a MW of 46 kDa, which indicates a trimer of ANGPTL4 (monomer 15.4 kDa). The UV trace representing protein absorbance at 280 nm is in *black* and MW is represented in *gray*. SEC, size-exclusion chromatography.

We next set out to determine the oligomeric state of N-terminal ANGPTL3 in peaks 1 and 2. Although a previous study showed that ANGPTL3 elutes from SEC across a broad peak as high-molecular-weight oligomers, the precise composition of these oligomers was not determined (32). Thus, both peaks 1 and 2 were collected, concentrated, and separately analyzed by SEC-MALS. These experiments revealed that peak 1 had a molecular weight of 151 kDa, which corresponds to a hexamer of ANGPTL3 (monomer 26.8 kDa) (Fig. 2*A*). The second peak had a molecular weight of 76 kDa, which corresponds to an ANGPTL3 trimer (Fig. 2*B*). It is intriguing that we also found that the ANGPTL3 hexamers and trimers do not interconvert. When we reinjected each separately concentrated peak onto SEC, we observed only a single peak on the second SEC run (Fig. 2, *A* and *B*), indicating that ANGPLT3 hexamers do not break down into trimers and trimers do not assemble into hexamers. It should also be noted that N-terminal ANGPTL3 does not have any cysteine residues, which indicates disulfides cannot account for the oligomerization. To further investigate the oligomeric state, we subjected ANGPTL3 to a denaturing purification and then refolded the protein and compared the elution from SEC with that of natively purified ANGPTL3. This revealed that following refolding ANGPTL3 forms trimers (Fig. S1). In order to ascertain the oligomeric state of ANGPTL3 in mammalian cells, we compared N-terminal ANGPTL3 produced in HEK-293 cells with *E. coli*-produced ANGPTL3 (Fig. S2). The HEK-293-produced ANGPTL3 elutes from SEC as a trimer. It eluted slightly before the *E. coli* ANGPTL3 trimer from SEC, which was expected owing to the addition of N-linked glycans in mammalian cells.

We also performed SEC-MALS on N-terminal ANGPTL4 and found ANGPTL4 elutes in a single peak with a molecular weight of 46 kDa, which corresponds to a trimer of ANGPTL4 (monomer 15.4 kDa, Fig. 2*C*).

### ANGPTL3 and ANGPTL4 have elongated and flexible structures

**Figure 3:**
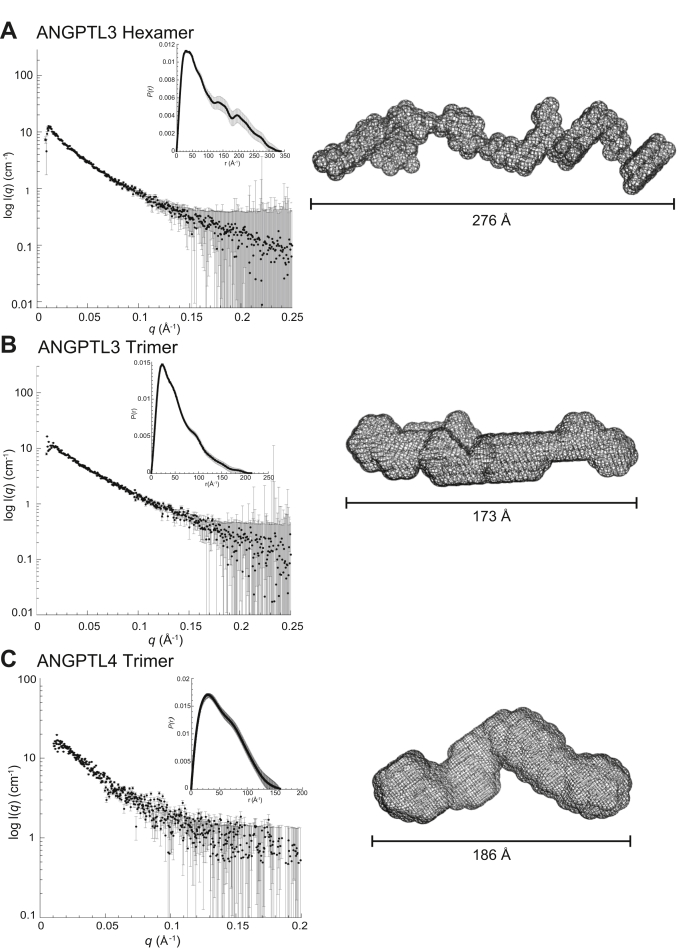
**ANGPTL3 and ANGPTL4 have elongated structures.** Size-exclusion chromatography–small-angle X-ray scattering scattering data as a log-linear plot with inset showing the real-space distance distribution and the filtered average *ab initio* models determined using DAMMIN for (*A*) ANGPTL3 hexamer (SASDJK8), (*B*) ANGPTL3 trimer (SASDJL8), and (*C*) ANGPTL4 (SASDJM8). Data deposited in the Small Angle Scattering Biological Data Bank (SASBDB) (61).

To further analyze the structural differences between ANGPTL3 and ANGPTL4, we carried out SEC-SAXS. ANGPTL3 hexamers and trimers were purified by SEC and then separately concentrated before being reinjected for SEC-SAXS (Fig. S3). Analysis of ANGPTL3 hexamers and trimers revealed elongated structural envelopes, with the trimer being about half the length of the hexamer (Fig. 3, *A* and *B*, Table S1). The length of molecules seen here is consistent with other coiled-coil proteins of similar size. ANGPTL4 was also found to have an elongated and highly flexible structure by SEC-SAXS (Fig. 3*C*, Table S1). In addition, both the ANGPTL3 hexamer and trimer and ANGPTL4 displayed characteristics of highly flexible proteins (Fig. S3). There is currently no structure of the N-terminal region of ANGPTL3, so we did not attempt to model in the predicted coiled-coil domains (33). Although these SAXS envelopes are of low resolution, they reveal that both ANGPTL3 and ANGPTL4 adopt elongated structures in solution and confirm that the SEC-MALS data accurately describe the oligomeric state of the proteins.

### ANGPTL3 and ANGPTL4 are potent noncompetitive inhibitors of LPL with VLDL as a substrate

**Figure 4:**
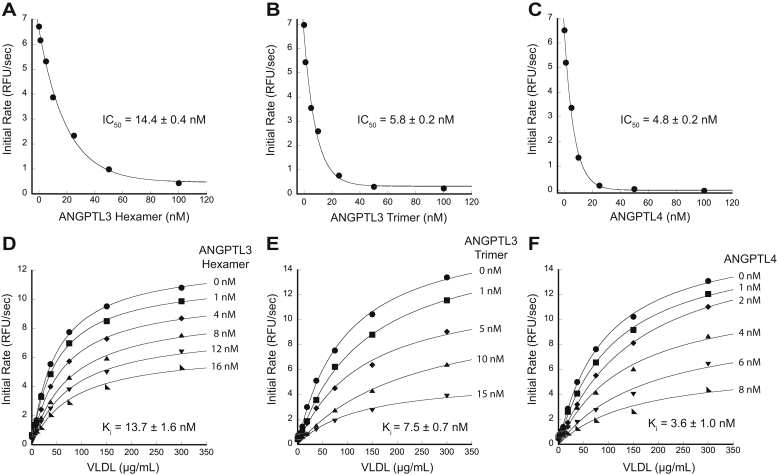
**ANGPTL3 and ANGPTL4 are both potent LPL inhibitors.** Representative IC_50_ curves were collected with 10 nM LPL, 200 μg/ml VLDL triglycerides, and varying amounts of (*A*) ANGPTL3 hexamer, (*B*) ANGPTL3 trimer, or (*C*) ANGPTL4. IC_50_ was calculated from three biological replicates and the error from the standard deviation. Data were fit with an exponential decay curve in KaleidaGraph and the IC_50_ determined from where the curve crossed 50% of the maximum LPL activity without inhibitor. The ANGPTL3 hexamer had the least potent IC_50_, whereas the IC_50_s for the ANGPTL3 trimer and ANGPTL4 were similar. Representative Michaelis–Menten curves were collected at 10 nM LPL with varying amounts of VLDL and (*D*) ANGPTL3 hexamer, (*E*) ANGPTL3 trimer, and (*F*) ANGPTL4 as indicated. Analysis of the data showed that the *V*_max_ decreased with increasing inhibitor concentration, and the *K*m did not change (Fig. S4). These results are consistent with a noncompetitive mechanism of inhibition for both ANGPTL3 and ANGPTL4. The reported *K*_i_ is the average of three biological replicates analyzed using a global noncompetitive fit, and the error is the standard deviation between replicates. LPL, lipoprotein lipase; VLDL, very-low-density lipoprotein.

We next analyzed ANGPTL3 and ANGPTL4 inhibition of LPL acting on one of its physiological substrates, VLDL. To do so, we adapted a previously published (27) method that detects nonesterified fatty acids (NEFAs) using an enzyme-coupled reaction in combination with Amplex UltraRed, a fluorescent reporter. This assay allows continuous readout of NEFA production so that initial rates of LPL hydrolysis can be measured with and without inhibitors. We first used this method to calculate the half-maximal inhibitory concentration (IC_50_) at a fixed amount of VLDL triglycerides (Fig. 4, *A*–*C*). This revealed that, when assayed on 10 nM LPL, the ANGPTL3 trimer (5.8 ± 0.2 nM) and ANGPTL4 trimer (4.8 ± 0.2 nM) share similar inhibition abilities, whereas the ANGPTL3 hexamer is a less potent inhibitor (14.4 ± 0.4 nM).

We used the IC_50_ data to guide design of assays varying both the inhibitor and VLDL concentration to collect a series of Michaelis–Menten curves for each inhibitor. We individually fit each Michaelis–Menten curve (Fig. 4, *D*–*F*) and further graphed the data by creating reciprocal plots, slope replots, and fractional velocity plots (Fig. S4). This allowed us to determine that both ANGPTL3 and ANGPTL4 show the characteristic traits of pure noncompetitive inhibition (34, 35). Following this determination, we used Mathematica to perform global noncompetitive fitting of the Michaelis–Menten curves to calculate the inhibition constant, K_i_. We averaged the resulting K_i_’s from three biological replicates to arrive at a K_i_ for each inhibitor. As expected from the IC_50_ data, ANGPTL3 hexamer had the least potent K_i_ (13.7 ± 1.6 nM). Both ANGPTL3 trimer (7.5 ± 0.7 nM) and ANGPTL4 (3.6 ± 1.0 nM) K_i_’s were more potent; however, they differed more than as calculated with the IC_50_. ANGPTL4 was twice as potent as the ANGPTL3 trimer, although both are effective inhibitors.

### Heparin binds to ANGPTL3 and prevents inhibition of LPL

**Figure 5:**
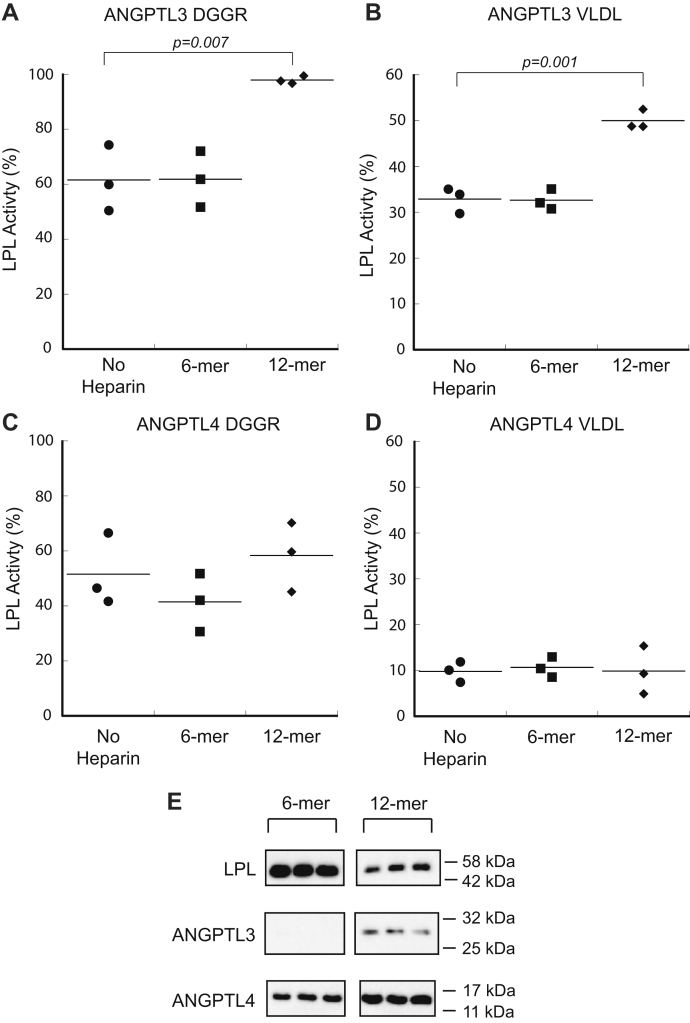
**Heparin effects LPL inhibition of ANGPTL3 and ANGPTL4.** The activity of LPL was determined in the presence of ANGPTL3 using both (*A*) DGGR and (*B*) VLDL as substrates. ANGPTL3 experiences a significant loss of inhibition when incubated with 12-mer heparin, but not 6-mer heparin for both substrates. LPL activity was also tested with ANGPTL4 using (*C*) DGGR and (*D*) VLDL substrates. ANGPTL4 is an effective inhibitor with addition of either heparin variant. *E*, biotinylated 6-mer and 12-mer were used to pull down human LPL, ANGPTL3, and ANGPTL4 and assessed *via* Western blot. Both LPL and ANGPTL4 bound to the 6-mer and 12-mer, whereas ANGPTL3 only showed binding to the 12-mer. Full blots are shown in Fig. S5. DGGR assays were performed with 2.5 nM LPL, 6 μM ANGPTL, 10 μg/ml heparin, and 10 μM DGGR. VLDL assays were performed with 10 nM LPL, 10 nM ANGPTL, 2 nM heparin, and 200 μg/ml VLDL triglycerides. Data were normalized to the activity of LPL without inhibitor present. Dots represent biological replicates; significance was determined by two-tailed Student’s *t* test. DGGR, 1,2-Di-O-lauryl-*rac*-glycero-3-glutaric acid 6′-methylresorufin ester; LPL, lipoprotein lipase; VLDL, very-low-density lipoprotein.

Previous work using 1,2-Di-O-lauryl-*rac*-glycero-3-glutaric acid 6′-methylresorufin ester (DGGR) as an LPL substrate demonstrated that ANGPTL3 inhibition of LPL is blocked by the presence of heparin, whereas ANGPTL4 inhibition is not (24). Given that the DNA-contaminated ANGPTL3 hexamer was a poor inhibitor of LPL, we hypothesized that both heparin and DNA might occupy a heparin-binding motif in ANGPTL3 (V61–G66), which is adjacent to the LPL inhibitory motif (I46–L57), and consequently prevent LPL inhibition (36). We tested this hypothesis by measuring the effects of two different-sized heparin oligosaccharides: hexasaccharide (6-mer) and dodecasaccharide (12-mer) on LPL inhibition by ANGPTL3 and ANGPTL4 (heparin structures shown in Fig. S5). We used two different substrates, DGGR for comparison with previous work (24) and VLDL as a natural substrate, and found that both substrates yielded the same results. ANGPTL3 effectively inhibited LPL in the presence of heparin 6-mer. However, ANGPTL3 inhibition of LPL was significantly decreased in the presence of heparin 12-mer (Fig. 5, *A* and *B*). By contrast, ANGPTL4 showed no impairment of inhibition from 6-mer or 12-mer heparin (Fig. 5, *C* and *D*). ANGPTL3 activity was also tested in the presence of mixed-molecular-weight heparin, and it significantly decreased ANGPTL3 inhibition similar to the 12-mer heparin (Fig. S5).

We hypothesized that the lack of ANGPTL3 inhibition in the presence of 12-mer was the result of heparin binding to ANGPTL3 and blocking inhibition by occluding ANGPTL3’s LPL-binding interface, rather than blocking the ANGPTL3-binding interface on LPL, as had previously been hypothesized (24). To confirm this, we synthesized biotinylated 6-mer and 12-mer. We analyzed LPL, ANGPTL3, and ANGPTL4 binding to both of these biotinylated heparin variants. Each protein was incubated with one of the biotinylated heparin variants and then pulled down using streptavidin-coated beads. The beads were pulled down and washed and the protein bound to the biotinylated heparin was analyzed by Western blot. We found that ANGPTL3 significantly bound to 12-mer but had little binding to 6-mer (Fig. 5*E*, Fig. S5). This supports that ANGPTL3 binding to 12-mer abolishes its ability to inhibit LPL, rather than heparin binding to LPL, given that LPL binds well to both 6-mer and 12-mer (Fig. 5*C*). ANGPTL4 has previously been shown to bind unfractionated heparin (37), and here we see it also binds to low-molecular-weight heparins. In spite of binding to ANGPTL4, the low-molecular-weight heparins did not affect ANGPTL4 inhibition of LPL (Fig. 5*E*).

## Discussion

Our comparison of ANGPTL3 with ANGPTL4 has clarified many puzzling aspects of ANGPTL3 and ANGPTL4 inhibition of LPL. Our discovery that ANGPTL3 copurifies with a DNA contamination could explain the differences in the reported potency of ANGPTL3 (24, 25, 26). Because ANGPTL3 is sensitive to both DNA and heparin inhibition, the presence of either in ANGPTL3 preparations could lead to inactive ANGPTL3. After ANGPTL3 was purified without contaminating DNA, we found that it was a potent inhibitor of LPL, even in the absence of ANGPTL8. This discovery has implications for the debate surrounding whether ANGPTL8 activates ANGPTL3 or vice versa. Because ANGPTL3 can inhibit LPL without ANGPTL8, but the reverse has not been seen (8, 10), we predict that ANGPTL8 likely works to activate ANGPTL3. The activation mechanism could involve a structural rearrangement or potentially the removal of inactivating agents, like heparin or DNA, from the active site of ANGPTL3.

This work presents the first structural characterization of the N-terminal domains of ANGPTL3 and ANGPTL4. Using both SEC-MALS and SEC-SAXS experiments, we found that N-terminal ANGPTL3 purifies in two separate oligomeric forms, hexamers and trimers, that do not spontaneously interconvert. However, when denatured and refolded, ANGPTL3 adopts a trimeric state indicating that the trimeric oligomer is likely the most stable arrangement of the protein. We also observed that ANGPTL3 produced in mammalian cells adopted a trimeric oligomer. ANGPTL4 was also observed to form trimers, which differs from previous reports that ANGPTL4 forms dimers, tetramers, or hexamers (20, 24). ANGPTL4 and ANGPTL3 both share a predicted coiled-coil structure and LPL inhibitory activity. Therefore, it is not unexpected that both proteins can form trimers. These assays used only the N-terminal domain of both proteins, purified from a recombinant source; therefore, it will be important to assess the oligomeric form of both proteins in the blood. Our data does align with recent characterizations of *in vivo* ANGPTL3/ANGPTL8 complexes that were identified by mass spectrometry to exist at a 3:1 ratio (11), which is consistent with trimeric ANGPTL3. However, the oligomeric state of the full-length ANGPTL3 and ANGPTL4 may also differ from those of the N-terminal constructs. Beyond the oligomeric state of the proteins, the SEC-SAXS structures of ANGPTL3 and ANGPTL4 revealed that both oligomers form elongated rod-like structures and likely have a high degree of flexibility.

The method by which ANGPTL4 inhibits LPL has long been debated in the field, and to a lesser extent the method of ANGPTL3 inhibition. It has become increasingly clear that a major factor in these differing results are the methods (assays, additives) used to determine the type of inhibition. Previously, we have shown that ANGPTL4 is a noncompetitive inhibitor using DGGR as a substrate (29). Fluorescent substrates like DGGR allow initial rate measurements, which is key for determining kinetic mechanisms. However, DGGR is a small molecule, and the inhibition values determined using DGGR are not likely to be reflective of the physiological values. Use of Amplex UltraRed allows collection of initial rate data using a natural substrate. We can now characterize the type of inhibition used by both ANGPTL3 and ANGPTL4 on human VLDL, rather than a nonphysiological substrate.

We found using the modified NEFA and Amplex assay that both ANGPTL3 and ANGPTL4 were noncompetitive inhibitors. This agrees with the results of previous DGGR assays that showed that ANGPTL4 is a noncompetitive inhibitor (29). It also agrees with the finding that ANGPTL3 is a reversible inhibitor (24). We used kinetic analysis to arrive at a mechanism of pure noncompetitive inhibition, the hallmarks of which are observing a decreasing V_max_ with increasing inhibitor concentrations and no change in K_m_ with inhibitor concentration (Fig. 4, *D* and *E*, Fig. S4). We also found that the reciprocal plots of the data intersect on the 1/[Substrate] axis and that the slope replots were linear (Fig. S4) (34). Noncompetitive inhibition indicates that ANGPTL3 and ANGPTL4 can bind to both LPL and substrate-bound LPL in a reversible manner. We believe that observing reversible inhibition with both DGGR and VLDL points in favor of this mode of inhibition being utilized *in vivo*. It will be important for the field to develop a standard assay protocol that can be used across laboratories to allow further comparisons of different additives and inhibitor combinations.

A previously known difference between ANGPTL3 and ANGPTL4 is their interaction with heparin. N-terminal ANGPTL3 possesses a heparin-binding site adjacent to its LPL inhibitory domain, whereas N-terminal ANGPTL4 binds heparin at an unidentified site (36, 37). It was initially hypothesized that ANGPTL3 inhibition of LPL was blocked by heparin binding to LPL (24). Our data show that LPL binds to both 6-mer and 12-mer heparin, but ANGPTL3 only binds to 12-mer heparin. Heparin-bound ANGPTL3 is not an effective LPL inhibitor, which we verified with both DGGR and VLDL substrates (Fig. 5, *A* and *B*). We also found that ANGPTL4 bound both 6-mer and 12-mer heparin, but neither affected its inhibition of LPL (Fig. 5, *C* and *D*). In the future, it would be interesting to explore if the presence of GPIHBP1 bound to LPL can also affect inhibitor binding in the presence of heparin. ANGPTL3’s loss of activity upon binding to heparin opens up new possible ways to block ANGPTL3 activity *in vivo*.

This work presents new insights into the structure and LPL inhibition of ANGPTL3 and ANGPTL4. It is clear that ANGPTL3 and ANGPTL4 share many global similarities, including elongated structures, trimeric oligomers, and noncompetitive inhibition. We have also identified an important difference, which is that the binding of ANGPTL3 to 12-mer heparin can block LPL inhibition. This knowledge will help in the development of effective therapeutics to enhance LPL activity.

## Experimental procedures

### ANGPTL3 cloning

The N-terminal domain of human ANGPTL3 (UniProt accession ID Q9Y5C1), encompassing residues S17 to R224, was cloned C terminally to a hexa-histidine tag and a TEV cleavage site. The resulting molecular weight of the purified protein was 26.8 kDa. The sequence was placed into a pETDuET vector (Novagen) at the multiple cloning site 1 (MCS1) (pETDuET-ANGPTL3). For human tissue culture expression, ANGPTL3 1 to 224 with a C-terminal hexa-histidine tag was placed in the pCDNA5-FRT-MCS expression vector.

### Purification of ANGPTL3 from *E. coli* (both with and without DNA)

pETDuET-ANGPTL3 was transformed into Bl21 (DE3) RIPL cells and grown in LB at 37 °C to an optical density of 0.6 at 600 nm. Protein expression was induced using 1 mM isopropyl β-D-1-thiogalactopyranoside (IPTG) and the cells were grown for 3 h at 37 °C before harvesting by centrifugation at 6700*g* for 30 min at 4 °C. Cells were resuspended in Binding Buffer (20 mM Tris-HCl pH 8, 500 mM NaCl, 5 mM Imidazole pH 8, 10% glycerol) on ice and flash frozen. Cells were thawed, and all subsequent steps were performed at 4 °C. Cells were lysed using an Emulsiflex C3 high-pressure homogenizer; then the lysate was clarified by centrifugation at 27,000*g* for 70 min. The supernatant was filtered with a 0.2-μm filter and applied to a Ni-NTA resin column equilibrated in Binding Buffer. Following binding, the column was washed in Binding Buffer followed by Wash Buffer 1 (20 mM Tris-HCl pH 8, 500 mM NaCl, 25 mM Imidazole pH 8, 10% glycerol), and Wash Buffer 2 (20 mM Tris-HCl pH 8, 300 mM NaCl, 60 mM Imidazole pH 8, 5% glycerol). Protein was eluted using Elution Buffer (20 mM Tris-HCl pH 8, 300 mM NaCl, 300 mM Imidazole pH 8, 5% glycerol). Fractions containing ANGPTL3 were pooled and dialyzed overnight into either MonoQ Buffer A (20 mM Tris-HCl pH 8, 100 mM NaCl, 5% glycerol) or SEC Buffer (20 mM Tris-HCl pH 8, 500 mM NaCl, 5% glycerol). For purifications using anion-exchange MonoQ chromatography, samples were loaded onto a MonoQ column using an AKTA explorer system. Protein was eluted *via* a linear gradient using MonoQ Buffer B (20 mM Tris-HCl pH 8, 1 M NaCl, 5% glycerol). Fractions containing ANGPTL3 were pooled and dialyzed overnight into SEC Buffer. SEC was performed using either a Superdex S-200 or Sephacryl S-300 column. The S-300 column provided more separation between the excluded volume and the first peak of ANGPTL3, although ANGPTL3 peak 1 and peak 2 were in the included volume of both columns. ANGPTL3 peak 1 and peak 2 were pooled separately and concentrated using 10 kDa cutoff Millipore filters. The final protein concentrations were determined by bicinchoninic acid assay, and DNA contamination was assessed by the 260:280 nm ratio with a Thermo Nanodrop.

For the ANGPTL3 denaturing purification, cells were grown as above and lysed by incubation for 1 h at 22 °C in 6 M guanidine hydrochloride (GuHCl). The lysate was clarified by centrifugation at 27,000*g* for 70 min at 22 °C. The supernatant was filtered with a 0.2-μm filter and applied to a Ni-NTA resin column equilibrated in Denaturing Binding Buffer (6 M GuHCl, 20 mM Tris-HCl pH 8, 200 mM NaCl, 5 mM Imidazole pH 8). A gradient mixer was used to rinse the column in a linear gradient from 100% Denaturing Binding Buffer to 100% Binding Buffer to refold the protein. The refolded ANGPTL3 was then eluted from the column as detailed above and assessed by SEC using a Superdex S-200 Increase column.

### Purification of ANGPTL3 from tissue culture

ANGPTL3 was stably integrated into Flp-In T-REx-293 human embryonic kidney cells (HEK-293) using the ANGPTL3 pCDNA5-FRT-MCS vector. Cells were maintained in Gibco DMEM with 10% fetal bovine serum (FBS), 1% penicillin/streptomycin, and 1% L-glutamine. Expression of ANGPTL3 was induced by the addition of expression media comprising Gibco Dulbecco's modified Eagle's medium with 1% FBS, 1% penicillin/streptomycin, 1% L-glutamine, and 2 μg/ml tetracycline. The expression medium was collected every 24 h and replaced with new expression medium for 5 days. The medium was flash frozen and stored at −80 °C. For purification, the medium was thawed, pooled, filtered, and bound to Ni Sepharose Excel beads at 4 °C. ANGPTL3 was then purified as detailed for *E. coli* expression.

### Purification of other proteins

N-terminal ANGPTL4 (UniProt accession ID Q9BY76) was cloned and purified as reported (29, 38). Bovine LPL (UniProt accession ID P11151) was purified from raw cow’s milk using heparin chromatography as reported (39). Furin-resistant human LPL (UniProt accession ID P06858) was purified as reported (40).

### SEC-MALS

SEC-MALS was performed using a 24-ml Sephadex S-200 column connected to a Wyatt DAWN HELEOS II light scattering instrument interfaced to an Agilent 1260 Infinity FPLC System, Wyatt T-rEX refractometer, and Wyatt dynamic light scattering module. For both ANGPTL3 and ANGPTL4 the S-200 column was equilibrated with buffer (20 mM Tris-HCl pH 8, 150 mM NaCl, 5% Glycerol) prior to injecting 90 μl of sample on to the column at 0.5 ml/min. SEC-MALS experiments were performed twice and calibration was confirmed with a bovine serum albumin (BSA) standard. Data were analyzed using the Wyatt ASTRA software package.

### SEC-SAXS ANGPTL3

SEC-SAXS of both ANGPTL3 oligomers was performed using the SIBYLS beamline 12.3.1 at the Advanced Light Source in Berkeley, California (41, 42, 43, 44). SEC was performed using an Agilent 1260 series HPLC with a Shodex Protein KW-804 analytical column at a flow rate of 0.5 ml/min in 20 mM Tris-HCl pH 7.5, 400 mM NaCl, 2% glycerol. Samples were shipped on ice and stored at 4 °C. Protein flowing off of the SEC was examined using SAXS at a sample-to-detector distance of 1.5 m, with λ = 1.03 Å incident light. This resulted in a q-range of 0.013 to 0.5 Å^−1^. Each exposure frame was 3 s. SEC-SAXS curves were initially analyzed by the SIBYLS beamline staff using ScÅtter (45). Data collection was repeated twice. Further information on the calibration of the SIBYLS beamline can be found in recent publications (46, 47).

### SEC-SAXS ANGPTL4

SAXS of ANGPTL4 was performed at the BioCAT beamline 18ID at the Advanced Photon Source (APS) (48) in Chicago, Illinois, with in-line SEC. The sample was loaded onto a WTC-015S8 column (Wyatt Technology) run by an Infinity II HPLC (Agilent Technologies) and run at 0.8 ml/min. After the SEC eluate passed through the UV monitor, it flowed through the SAXS flow cell, which consists of a 1.5-mm ID quartz capillary with 10-μm walls. The scattering intensity was recorded using a Pilatus3 1M (Dectris) detector, which was placed 3.5 m from the sample, giving us access to a q-range of 0.004 to 0.4 Å^−1^. Exposures, 0.5 s, were acquired every 2 s during elution, and data were reduced using BioXTAS RAW 1.4.0 (49). Buffer blanks were created by averaging regions flanking the elution peak and subtracted from exposures selected from the elution peak to create the I(q) *versus* q curves used for subsequent analyses. ANGPTL4 was also analyzed by traditional SAXS at the 12-ID-B beamline at APS.

### SAXS data analysis

The ATSAS software package was used for SAXS data analysis (50). PRIMUS was used to analyze the averaged and background subtracted SAXS data for Guinier range with Autorg (51, 52). P(r) was determined empirically with the help of GNOM (53). With these input parameters (Table S1), DAMMIF was run using the slow setting for 20 runs, assuming P1 symmetry and unknown shape (54). The results of DAMMIF runs were fed into the DAMAVER package and assessed for normalized spatial discrepancy (NSD) to determine how similar the 20 models were (55). The resulting damstart.pdb envelope from DAMAVER was used as the initial model for a final slow DAMMIN refinement with P1 symmetry, unknown shape, and all atoms unfixed (56). The fit of final structural envelopes was validated using CorMap (57).

### Amplex UltraRed LPL activity assays

Activity assays were performed using a final concentration of 10 nM bovine LPL. LPL was incubated with ANGPTL3 or ANGPTL4 for 10 min at 22 °C in phosphate buffered saline (PBS) with FBS prior to adding substrate for a final concentration in the well of 0.2× PBS (27 mM NaCl, 0.5 mM KCl, 2 mM Na_2_HPO_4_, 0.36 mM KH_2_PO_4_) and 2% FBS. Human VLDL (Athens Research & Technology, 12-16-221204) was used as a natural substrate, and the liberation of free fatty acid was detected using an enzyme-coupled reaction combined with a fluorescent reporter, Amplex UltraRed (amplex) (ThermoFisher, A36006).

The enzyme-coupled reaction is based on a colorimetric nonesterified fatty acid (NEFA) assay that we have previously used (58). The combination of NEFA assay and amplex was initially reported by Nimonkar *et al.* (27), and we adapted their method to collect initial rate inhibition curves. NEFA assay enzymes, VLDL, and amplex were added to the LPL and ANGPTL sample to begin the reaction with final concentrations in the well of 133 mM KPO_4_ pH 7.5, 150 mM NaCl, 3.3 mM MgCl_2_, 4.4 mM adenosine triphosphate, 1 mM Coenzyme A (CoA), 0.1 U/ml Acetyl-CoA synthetase (ACS), 6 U/ml horseradish peroxidase, 5 U/ml Acetyl-CoA oxidase (ACO), 0.2 mg/ml fatty acid–free BSA, 0.05 mM amplex, and 200 μg/ml triglycerides in human VLDL. For Michaelis–Menten curves, the amount of VLDL was varied by serial dilution. For assays including heparin, 2 nM 6-mer, 12-mer, or mixed-molecular-weight heparin was incubated with the LPL/ANGPTL mix for 10 min. Assays were conducted in a black-walled, 96-well plate. Immediately following the addition of the substrate, fluorescence was monitored using a M5 Spectramax plate reader at 37 °C, excitation at 529 nm and emission at 600 nm, with a 590-nm cutoff filter. The initial rate was determined from the first 180 s of the reaction. Three biological replicates were conducted for each assay. Data were graphed in KaleidaGraph, and significance was assessed using a two-tailed Student’s *t* test. Individual Michaelis–Menten curves were fit in KaleidaGraph. Global noncompetitive fitting was performed in Mathematica.

### DGGR heparin LPL activity assays

Activity assays were performed using a final concentration of 2.5 nM bLPL diluted in LPL assay buffer, which has a final concentration of 20 mM Tris-HCl pH 8.0, 150 mM NaCl, 0.2% fatty acid–free BSA, and 1 mM sodium deoxycholate. ANGPTL3 or ANGPTL4 were diluted to the necessary concentrations using SEC Buffer. LPL was mixed with ANGPTL and incubated in a black-walled, 96-well plate at 22 °C for 10 min. Fluorescent substrate 1,2-di-O-lauryl-rac-glycero-3-glutaric acid-(6’-methylresofurin) ester (DGGR) (Sigma) in 0.01% Anzergent 3 to 16 (Anatrace) was added immediately before assaying activity at 37 °C as described (29). Data were collected using the same method as the VLDL assays, and background was corrected using samples without LPL. For assays with heparin, 10 μg/ml of 6-mer, 12-mer, or mixed-molecular-weight heparin was added to the LPL/ANGPTL mix and allowed to incubate at 22 °C for 10 min. Data for LPL with ANGPTL3 or ANGPTL4 and heparin were normalized to LPL with heparin (without ANGPTL3 or ANGPTL4). Significance was determined by a two-tailed Student’s *t* test.

### Synthesis of heparin and biotinylated heparin oligosaccharides

Heparin 6-mer and 12-mer were synthesized according to a published chemoenzymatic method (59). More information is available in the supporting methods. The products were purified by anion-exchange chromatography using a Q-Sepharose column. Nuclear magnetic resonance and electrospray ionization mass spectrometry matched published data for 6-mer and 12-mer. Biotinylation of heparin oligosaccharides was performed using previously published methods (60). The final product was purified by DEAE-HPLC to generate biotinylated 6-mer and 12-mer. HPLC and MS results match the published report for these compounds.

### Biotinylated heparin pull-down and Western blots

First, 5 pmol of each protein (furin-resistant hLPL or ANGPTL3 hexamer or ANGPTL4) was incubated with 275 pmol of each biotinylated heparin (6-mer or 12-mer) in pull-down buffer (PBS, 0.1% fatty acid–free BSA, 0.01% Triton X-100). As a negative control, each protein was mixed with an equal volume of pull-down buffer instead of heparin. The samples were rotated at 4 °C for 30 min. Each sample was mixed with cleaned Streptavidin M-280 Dynabeads (Invitrogen). Samples with beads were rotated at 4 °C for 30 min. The beads were pulled down using a magnet, and the supernatant was removed by pipet. The beads were then washed with pull-down buffer three times. After removing the final supernatant, the beads were resuspended in 10% SDS to denature the protein and heated to 95 °C. The reactions were analyzed using Western blot. Proteins were separated by 14% SDS-PAGE, transferred to 0.22-μm polyvinylidene difluoride membrane, and blocked with 5% non-fat milk in TBS-T (20 mM Tris-HCl pH 7.6, 150 mM NaCl, and 0.01% Tween 20). LPL and ANGPTL3 were probed with a mouse anti-histidine tag antibody (Bio-Rad) using a 1:5000 dilution, and protein was detected with a horseradish peroxidase–conjugated goat anti-mouse antibody (Southern Biotech) using a 1:5000 dilution. ANGPTL4 was probed with a polyclonal rabbit anti-ANGPTL4 antibody (BioVendor) using a 1:5000 dilution and detected with a horseradish peroxidase–conjugated donkey anti-rabbit antibody (Southern Biotech) using a 1:5000 dilution. Western blots were developed as previously described (40).

## Data availability

Data for SAXS structural studies were deposited in the SASBDB under ascension codes SASBJK8, SASDJL8, and SASDJM8.

## Accension codes

Human ANGPTL3 - UniProt accession ID Q9Y5C1

Human ANGPTL4 - UniProt accession ID Q9BY76

Bovine LPL - UniProt accession ID P11151

Human LPL - UniProt accession ID P06858

## Conflict of interest

The authors declare that they have no conflicts of interest with the contents of this article.
