# Supplementary material for: Comparison of angiopoietin-like protein 3 and 4 reveals structural and mechanistic similarities
Source: J Biol Chem. 2021 Jan 20;296:100312. doi: 10.1016/j.jbc.2021.100312 (PMC7949051; doi:10.1016/j.jbc.2021.100312)
Supplement: Supplemental Figures and Tables [file mmc1.pdf]

SUPPORTING INFORMATION

Comparison of angiopoietin-like protein 3 and 4 reveals structural and mechanistic similarities

Kathryn H. Gunn[1], Aspen R. Gutgsell[1], Yongmei Xu[2], Caitlin V. Johnson[3], Jian Liu[2], Saskia B. Neher[1*]

[1]Department of Biochemistry and Biophysics, University of North Carolina, Chapel Hill, North Carolina, 27599, USA
[2]Division of Chemical Biology and Medicinal Chemistry, Eshelman School of Pharmacy, University of North Carolina, Chapel Hill, North Carolina, 27599, USA
[3]Department of Chemistry, University of North Carolina, Chapel Hill, North Carolina, 27599, USA

* Corresponding author: Saskia Neher - neher@email.unc.edu

Running Title: Structural and Mechanistic Similarities of ANGPTL3 and 4

Supporting Information Pages – 12 pages
Supporting Methods – 1 page
Supporting Figures – 5 figures
Supporting Tables – 1 table
Supporting References – 2 pages

SUPPORTING METHODS

Synthesis of heparin oligosaccharides

Both 6-mer and 12-mer were synthesized according to the chemoenzymatic method published previously (1). Briefly, heparosan synthase-2 (PmHS2) from *Pasteurella multocida* was used in conjunction with urine diphosphate (UDP) sugars to elongate the monosaccharide, glucuronic acid – p-nitrophenol (GlcA-pNP), to appropriately sized backbones. The backbone was then subjected to the modification of N-sulfotransferase (NST), C5-epimerase (C5-epi), 2-O-sulfotransferase (2-OST), 6-O-sulfotransferase (6-OST), and 3-O-sulfotransferase isoform 1 (3-OST-1). There were seven major steps involved in the overall synthesis, including an elongation step to add UDP-N-trifluoroacetyl glucosamine (GlcNTFA), an elongation step to add GlcA, a detrifluoroacetylation/N-sulfation step, epimerization, 2-O-sulfation, 6-O-sulfation, and 3-O-sulfation. These steps were repeated to prepare the 6-mer and 12-mer products (Supporting Figure 5A). The products were purified by anion-exchange chromatography using a Q-Sepharose column. The structures of the products were proven by nuclear magnetic resonance (NMR) and mass spectrometry (MS).

Synthesis of Biotinylated heparin oligosaccharides

Biotinylation of low molecular weight heparin was performed, using previously published methods (2). The reaction was started with 40 mg of p-nitrophenol (pNP) tagged 6-mer and 12-mer, mixed with 3 mg of palladium on carbon (Pd/C). This was dissolved in 20 mM NaOAc, pH 5.0 in a total volume of 10 mL, then the reaction mixture was vacuumed and refilled with H_2 three times. The reaction was then incubated at room temperature (22°C) for 4 hrs. After that, it was filtered to remove charcoal. The filtered solution was adjusted to pH 8.5 using 500 mM Na_2HPO_4. Succinimidyl 6-azidohexanoate (8 molar equivalents of 6-mer and 12-mer) was added and incubated at 37 °C overnight. Reaction was purified by DEAE- high pressure liquid chromatography (HPLC) column to generate azido-tagged 6-mer and 12-mer. PBS (pH 7.4) buffer was bubbled using N_2 for 5 min before preparing the sample solution, which had a final concentration of 0.1 M $CuSO_4$, 0.1 M Tris(3-hydroxypropyltriazolylmethyl)amine (THPTA) (Sigma), 0.15 M sodium ascorbate, 0.01 M azido tagged 6-mer or 12-mer and 0.02 M biotin-PEG$_4$-alkyne (Sigma). To create the sample solution, 2 mL THPTA and 400 µl $CuSO_4$ were vortexed together, then 800 µl sodium ascorbate, 1 mL 6-mer or 12-mer, and 1 mL biotin-PEG$_4$-alkyne (Sigma) was added and bubbled using N_2 for 2 min. The sample solution was incubated at 37 °C overnight. This reaction was purified by DEAE-HPLC column to generate biotinylated 6-mer and 12-mer. The reactions were monitored using HPLC and MS.

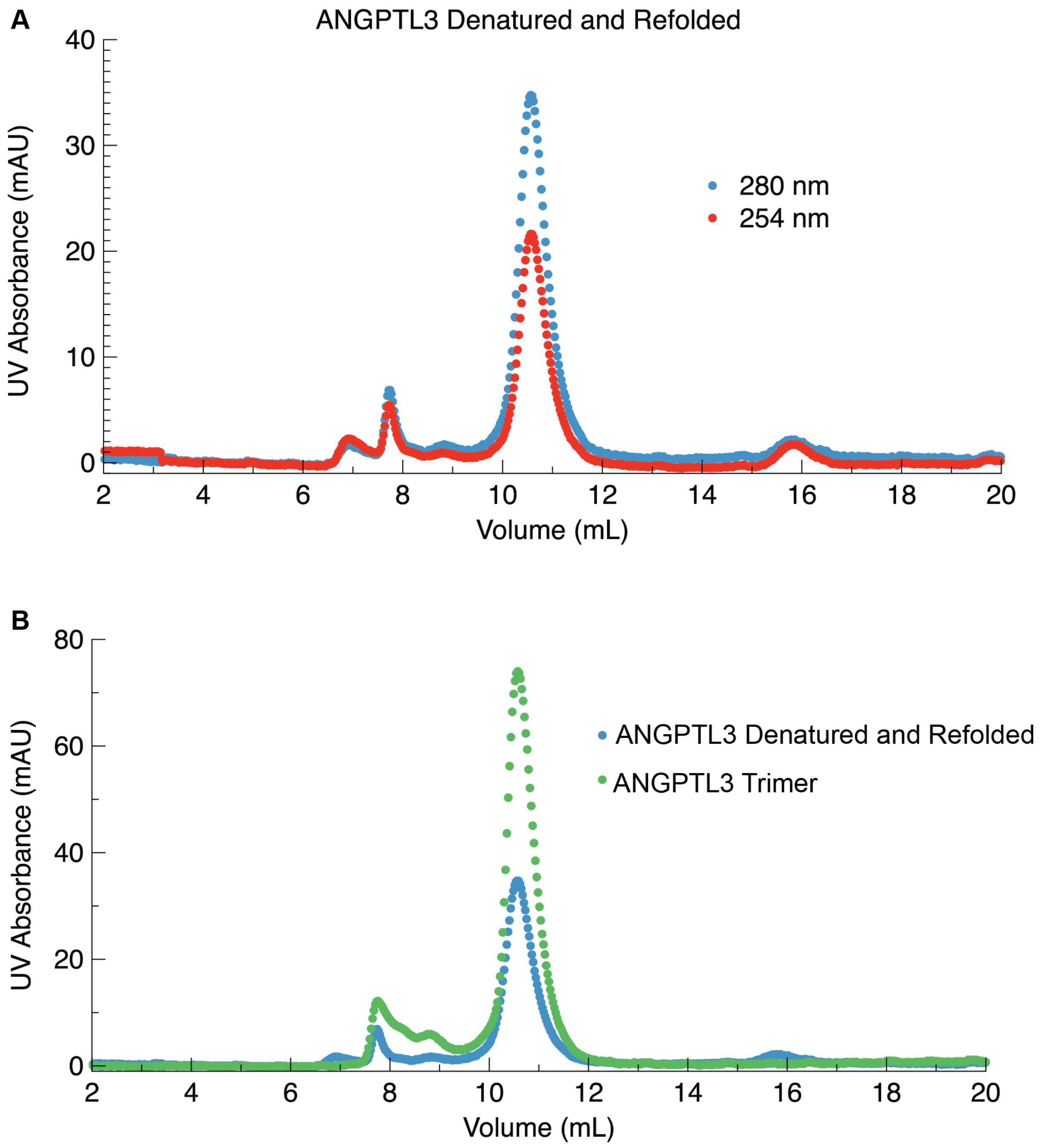

Supporting Figure 1

ANGPTL3 expressed in *E. coli* was denatured and bound to Nickel Sepharose beads. ANGPTL3 was then refolded on the Nickel beads and eluted. (A) This denatured and refolded ANGPTL3 was run on SEC using an S200 Increase column. (B) When compared to the 280 nm absorbance for ANGPTL3 trimer, which has not been denatured, the refolded 280 nm absorbance exactly overlaps and is, therefore, refolding as a trimer.

°

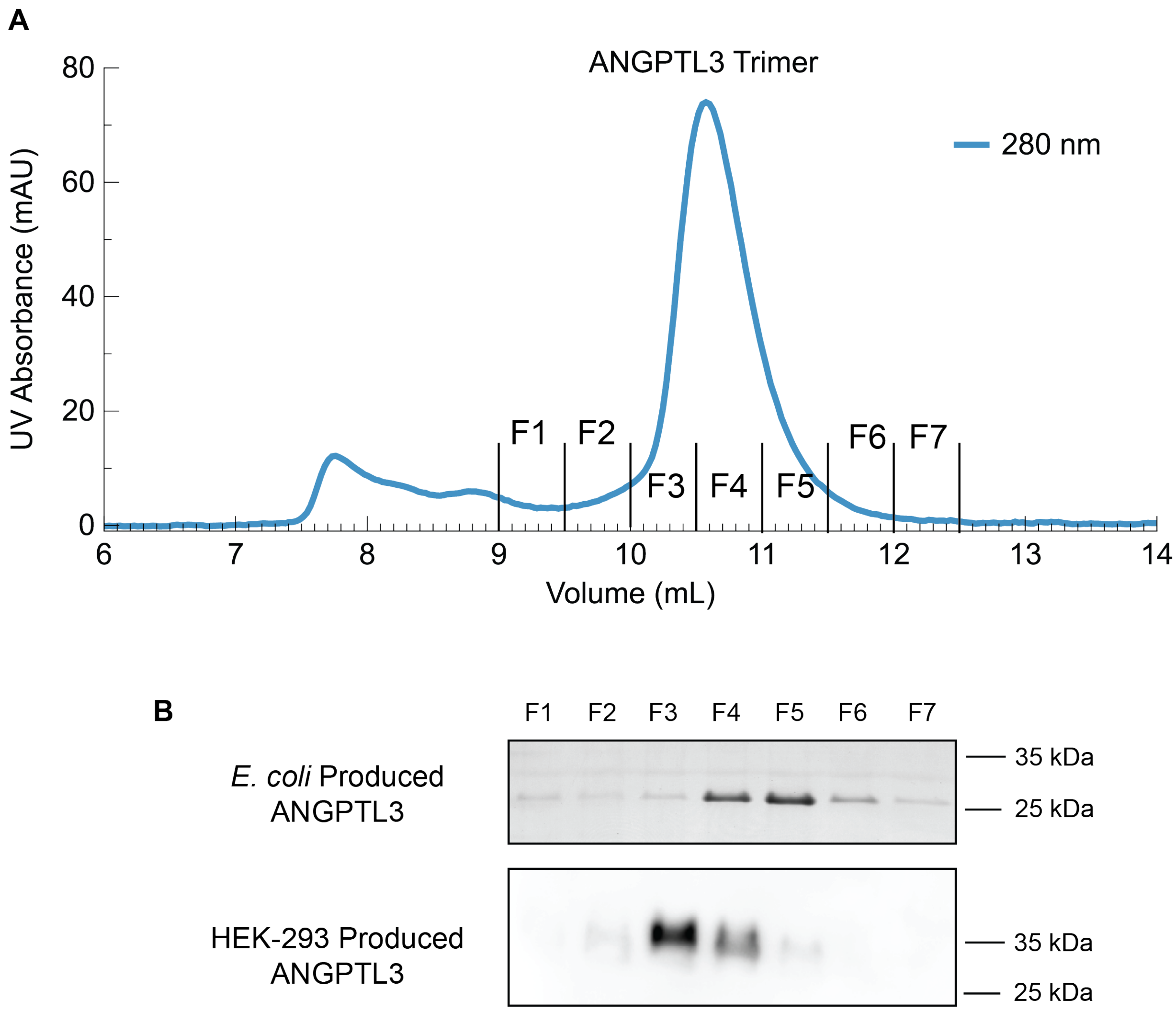

Supporting Figure 2

(A) The *E. coli* expressed ANGPTL3 trimer was injected onto an S200 Increase SEC column and the UV absorbance at 280 nm was used to determine the fractions ANGPTL3 eluted in (fractions labeled F1, F2, etc). (B) The *E. coli* expressed ANGPTL3 fractions were assessed by SDS-PAGE and Coomassie stained (upper gel). N-terminal ANGPTL3 was expressed and secreted from HEK-293 cells and then purified from the media. HEK-293 produced ANGPTL3 was then injected identically to the *E. coli* produced ANGPTL3 onto the S200 Increase SEC column. Due to the low concentration of HEK-293 produced ANGPTL3, fractions were assessed by western blot (lower blot). The HEK-293 produced ANGPTL3 elutes slightly before the ANGPTL3 trimer. The mammalian produced ANGPTL3 contains an N-linked glycosylation, which is not present in the *E. coli* produced ANGPTL3, which you can observe by the larger molecular weight seen in the western blot. Therefore, the earlier elution is likely due to the presence of glycosylation's, which add ~12 kDa in total to molecular weight to the complex. This indicates that tissue culture produced ANGPTL3 is a trimer. Data was graphed in DataGraph.

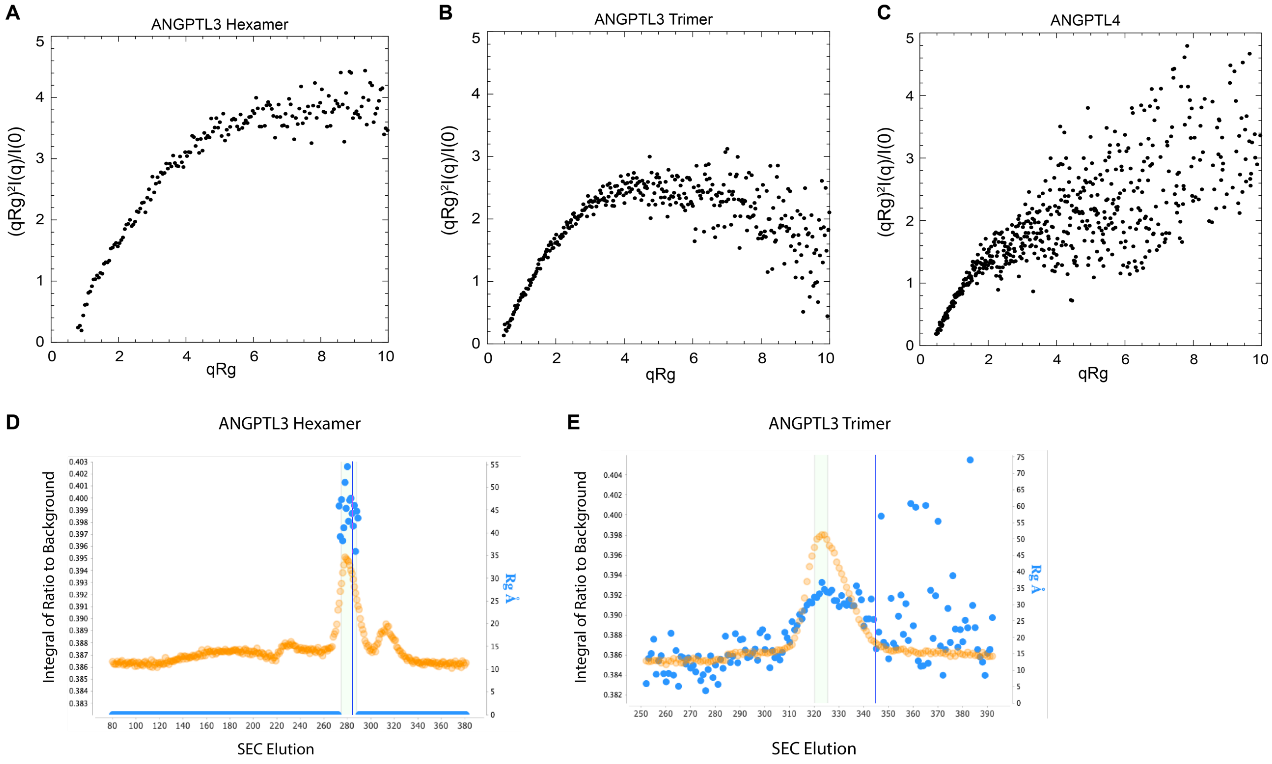

Supporting Figure 3

ANGPTL3 and ANGPTL4 form highly flexible and elongated structures. Dimensionless Kratky plots reveal a high degree of flexibility in (A) ANGPTL4, (B) ANGPTL3 hexamer, and (C) ANGPTL3 trimer characteristic of unfolded or intrinsically disordered flexible proteins. Size exclusion chromatography traces taken during ANGPTL3 SEC-SAXS at the SIBYLS beamline for (D) ANGPTL3 Hexamer and (E) ANGPTL3 Trimer. ANGPTL3 used for SEC-SAXS was previously SEC purified and each peak separately concentrated before being reinjected for SEC-SAXS.

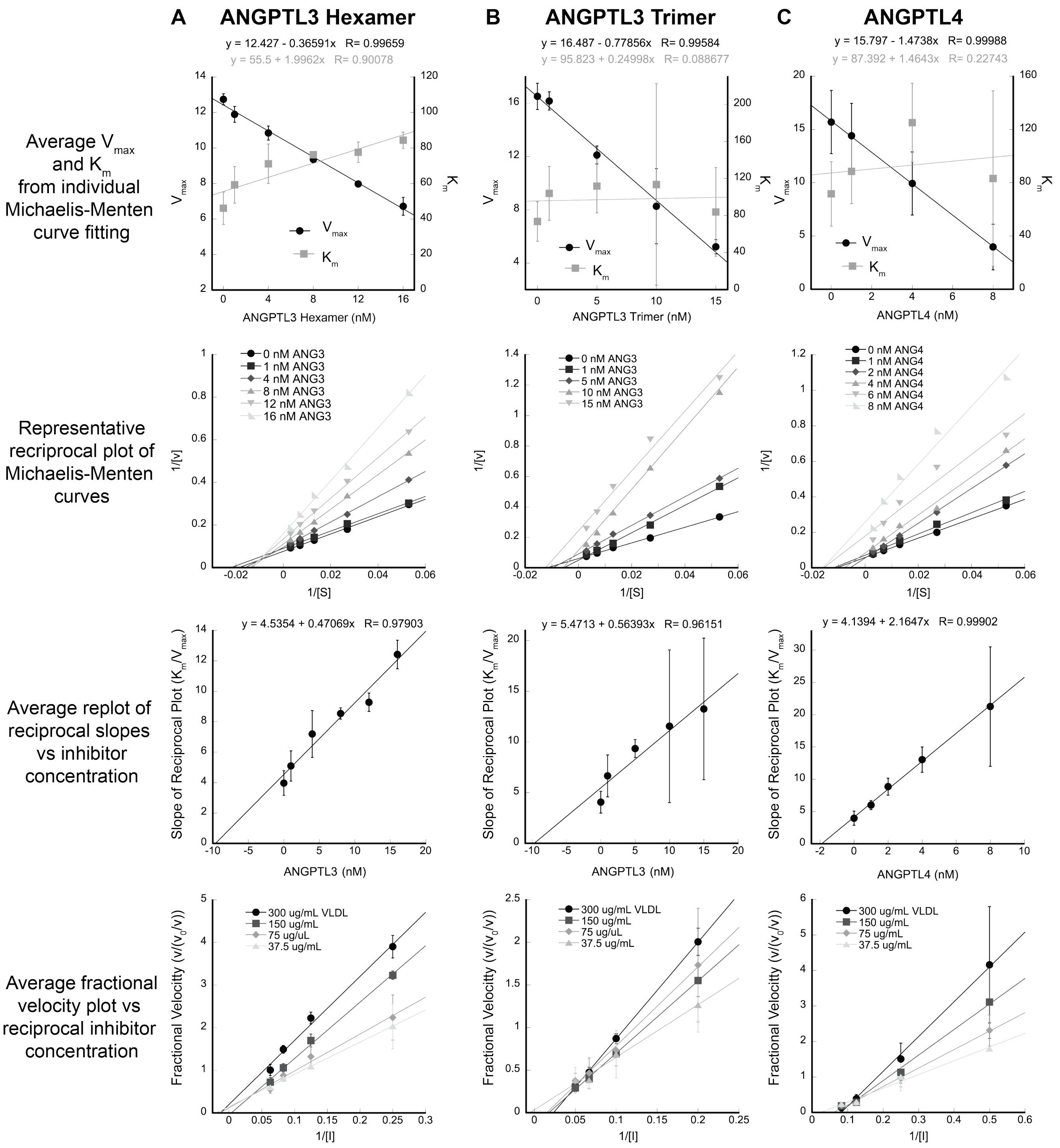

Supporting Figure 4

In-depth kinetic analysis to determine inhibition type (A) ANGPTL3 hexamer, (B) ANGPTL3 trimer, (C) ANGPTL4. With increasing inhibitor concentrations V_{max} decreases and K_m does not show an increasing or decreasing trend for ANGPTL3 trimer and ANGPTL4 trimer, characteristic of non-competitive inhibition (3). ANGPTL3 hexamer has an increasing trend in K_m, and a clear decreasing V_{max}. However, further analysis reveals characteristics of non-competitive inhibition

for the ANGPTL3 hexamer. Reciprocal plots of all inhibitors Michaelis-Menten curves intersect on the 1/[S] axis ([S] is the VLDL triglyceride concentration), characteristic of non-competitive inhibition (3). Replot of the slopes from the reciprocal plots (the slopes of the lines fit to each inhibitor concentration with units (K_m/V_{max})) are linear, indicative of pure non-competitive inhibition (3). The fractional velocity plots intersect at ~0, which is characteristic of complete inhibition (4). On the y-axis of fractional velocity plot is $v/(v_0 - v)$ (v is initial rate when inhibitor is present and v_0 is the rate without inhibitor). The x-axis is the reciprocal of the ANGPTL concentration (1/[Inhibitor]). Error bars are standard deviation from 3 biological replicates. Linear fits performed in KaleidaGraph.

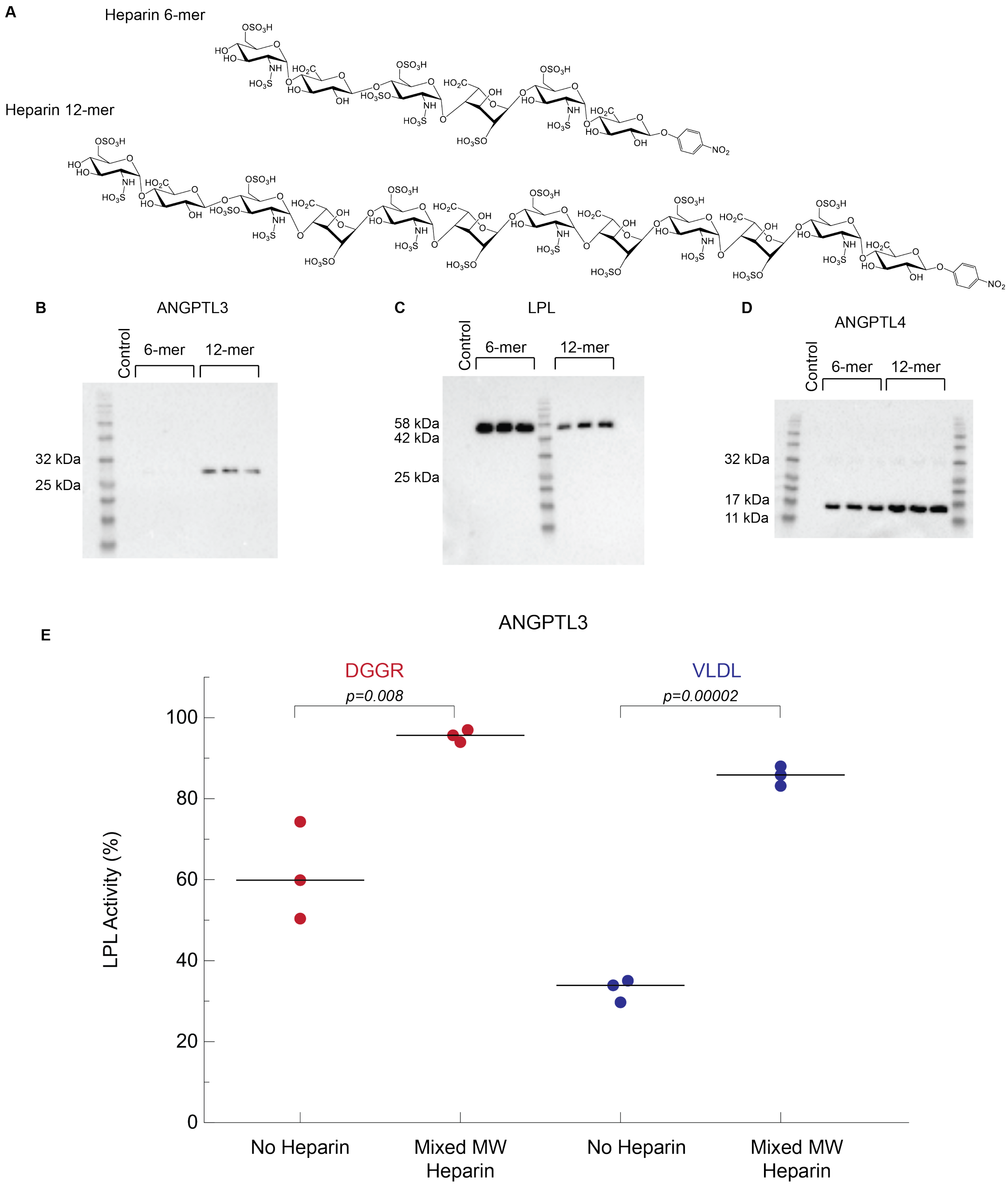

Supporting Figure 5

(A) Structures of heparin 6-mer and 12-mer used in activity assays. Full blots of biotinylated heparin pull-down of (B) ANGPTL3, (C) human furin-resistant LPL or (D) ANGPTL4 seen in Figure 5C. The protein molecular weight ladder is labeled to the left of the gel. (E) ANGPTL3 inhibition of LPL was tested in the presence mixed molecular weight (MW) heparin (which has a median MW of 18,000 kDa). Using either DGGR or VLDL as a substrate, ANGPTL3 inhibition of LPL is significantly diminished by mixed MW heparin. Data was graphed in DataGraph.

Sample details		
	ANGPTL3	ANGPTL4
Organism	*H. sapiens*	*H. sapiens*
Source (Catalogue No. or reference)	*E. coli*	*E. coli*
Description: sequence (including Uniprot ID + uncleaved tags), bound ligands/modifications, *etc.*	N-term hexahistidine tag + TEV cleavage site + Q9Y5C1 (17-224)	Q9BY76 (26-161)
Extinction coefficient ε (A_{280}, 0.1% w/v)	0.277	0.357
Partial specific volume $\bar{v}$ (cm^3 g^{-1})	0.731	0.733
Particle contrast from sequence and solvent constituents, $\Delta\bar{\rho}$ (cm^{-2})	2.842 (12.414-9.571)	1.902 (12.382-10.479)
Molecular mass *M* from chemical composition (Da)	26847.07	15397.35
SEC-SAXS column<br>Loading volume/concentration ($mg\ ml^{-1}$)<br>Injection volume (μl)<br>Flow rate ($ml\ min^{-1}$)	Shodex Protein KW-804<br>5.6 $mg\ ml^{-1}$ (Hexamer)<br>6.15 $mg\ ml^{-1}$ (Trimer)<br>80 μL<br>0.5 $ml\ min^{-1}$	Wyatt WTC-030S5<br>5 $mg\ ml^{-1}$<br><br>300 μL<br>0.8 $ml\ min^{-1}$
Solvent composition and source	20 mM Tris-HCl pH 7.5, 400mM NaCl, 2% Glycerol	50mM Tris-HCl pH 7.4, 300mM NaCl, 100mM Betaine, 500mM Arginine
SAXS data collection parameters		
Instrument/data processing	Advanced Light Source SIBYLS Beamline 12.3.1 (5,6)	Argonne National Laboratory Advanced Photon Source Undulator 18-ID (BioCAT) Beamline (7)
Wavelength (Å)	0.73 – 2.5	1.03
Beam size (μm)	5000 x 500	35 x 135
q-measurement range ($Å^{-1}$)	0.013 – 0.5	0.010 – 0.4
Exposure time	Continuous 3 s data-frame measurement of SEC elution	Continuous 0.5 s data-frame measurements of SEC elution
Sample configuration	SEC-SAXS	SEC-SAXS
Sample temperature (ºC)	25	25
Software employed for SAS data reduction, analysis and interpretation		
SAXS data reduction	Solvent subtraction *ScÅtter* (8)	Solvent subtraction *PRIMUSqt* (9,10)
Calculation of ε from sequence	*ProtParam* (11)	*ProtParam* (11)
Calculation of $\Delta\bar{\rho}$ and $\bar{v}$ values from chemical composition	*MULCh 1.1* (12)	*MULCh 1.1* (12)

Basic analyses: Guinier, *P*(*r*), scattering particle volume (*e.g.* Porod volume V_P or volume of correlation V_c)	*PRIMUSqt* (9,10)		*PRIMUSqt* (9,10)
Shape/bead modelling	*DAMMIF* (13) and *DAMMIN* (14)		*DAMMIF* (13) and *DAMMIN* (14)
Molecular graphics	*Pymol v1.8.2.2* Mac		*Pymol v1.8.2.2* Mac
Structural parameters			
Guinier Analysis	ANGPTL3 Hexamer	ANGPTL3 Trimer	ANGPTL4
$I(0)$ (cm^{-1})	13.89	13.34	18.28
R_g (Å)	56.41	46.58	46.45
q-range (Å^{-1})	0.013 - 0.026	0.013 - 0.025	0.015 - 0.027
Coefficient of correlation, R^2	0.93	0.78	0.79
P(*r*) analysis			
$I(0)$ (cm^{-1})	19.88	13.25	17.50
R_g (Å)	92.74	49.0	45.31
d_{max} (Å)	340	215	160
q-range (Å^{-1})	0.012 - 0.22	0.013 – 0.25	0.013 – 0.17
Shape modelling results			
	ANGPTL3 Hexamer	ANGPTL3 Trimer	ANGPTL4
q-range for fitting (Å^{-1})	0.012 - 0.22	0.013 - 0.25	0.013 - 0.17
Symmetry/anisotropy assumptions	P1, none	P1, none	P1, none
χ^2 value	1.19	2.3	0.8
P value	0.16	0.33	0.89
Data and model deposition IDs			
	ANGPTL3 Hexamer	ANGPTL3 Trimer	ANGPTL4 Trimer
SASBDB Code (15)	SASDJK8	SASDJL8	SASDJM8

Supporting Table 1

Detailed information for ANGTPL3 hexamer, ANGPTL3 trimer, and ANGPTL4 SEC-SAXS analysis including: sample details, data acquisition, data analysis, modelling fitting and software utilized. Table adapted from Trewhella *et al.* (16).

Supporting References

1. Xu, Y., Chandarajoti, K., Zhang, X., Pagadala, V., Dou, W., Hoppensteadt, D. M., Sparkenbaugh, E. M., Cooley, B., Daily, S., Key, N. S., Severynse-Stevens, D., Fareed, J., Linhardt, R. J., Pawlinski, R., and Liu, J. (2017) Synthetic oligosaccharides can replace animal-sourced low-molecular weight heparins. *Sci Transl Med* **9**
2. Miller, C. M., Xu, Y., Kudrna, K. M., Hass, B. E., Kellar, B. M., Egger, A. W., Liu, J., and Harris, E. N. (2018) 3-O sulfation of heparin leads to hepatotropism and longer circulatory half-life. *Thromb Res* **167**, 80-87
3. Segel, I. H. (1975) *Enzyme kinetics : behavior and analysis of rapid equilibrium and steady state enzyme systems*, Wiley, New York
4. Yoshino, M. (1987) A graphical method for determining inhibition parameters for partial and complete inhibitors. *Biochem J* **248**, 815-820
5. Classen, S., Hura, G. L., Holton, J. M., Rambo, R. P., Rodic, I., McGuire, P. J., Dyer, K., Hammel, M., Meigs, G., Frankel, K. A., and Tainer, J. A. (2013) Implementation and performance of SIBYLS: a dual endstation small-angle X-ray scattering and macromolecular crystallography beamline at the Advanced Light Source. *J Appl Crystallogr* **46**, 1-13
6. Dyer, K. N., Hammel, M., Rambo, R. P., Tsutakawa, S. E., Rodic, I., Classen, S., Tainer, J. A., and Hura, G. L. (2014) High-throughput SAXS for the characterization of biomolecules in solution: a practical approach. *Methods Mol Biol* **1091**, 245-258
7. Fischetti, R., Stepanov, S., Rosenbaum, G., Barrea, R., Black, E., Gore, D., Heurich, R., Kondrashkina, E., Kropf, A. J., Wang, S., Zhang, K., Irving, T. C., and Bunker, G. B. (2004) The BioCAT undulator beamline 18ID: a facility for biological non-crystalline diffraction and X-ray absorption spectroscopy at the Advanced Photon Source. *J Synchrotron Radiat* **11**, 399-405
8. Forster, S., Apostol, L., and Bras, W. (2010) Scatter: software for the analysis of nano- and mesoscale small-angle scattering. *Journal of Applied Crystallography* **43**, 639-646
9. Franke, D., Petoukhov, M. V., Konarev, P. V., Panjkovich, A., Tuukkanen, A., Mertens, H. D. T., Kikhney, A. G., Hajizadeh, N. R., Franklin, J. M., Jeffries, C. M., and Svergun, D. I. (2017) ATSAS 2.8: a comprehensive data analysis suite for small-angle scattering from macromolecular solutions. *J Appl Crystallogr* **50**, 1212-1225
10. Petoukhov, M. V., Franke, D., Shkumatov, A. V., Tria, G., Kikhney, A. G., Gajda, M., Gorba, C., Mertens, H. D., Konarev, P. V., and Svergun, D. I. (2012) New developments in the ATSAS program package for small-angle scattering data analysis. *J Appl Crystallogr* **45**, 342-350
11. Gasteiger, E., Hoogland, C., Gattiker, A., Duvaud, S., Wilkins, M. R., Appel, R. D., and Bairoch, A. (2005) Protein identification and analysis tools on the ExPASy server. in *The Proteomics Protocols Handbook* (Walker, J. M. ed.), Humana Press. pp pp. 571-607
12. Whitten, A. E., Cai, S. Z., and Trewhella, J. (2008) MULCh: modules for the analysis of small-angle neutron contrast variation data from biomolecular assemblies. *Journal of Applied Crystallography* **41**, 222-226
13. Franke, D., and Svergun, D. I. (2009) DAMMIF, a program for rapid ab-initio shape determination in small-angle scattering. *J Appl Crystallogr* **42**, 342-346
14. Svergun, D. I. (1999) Restoring low resolution structure of biological macromolecules from solution scattering using simulated annealing. *Biophys J* **76**, 2879-2886

15. Kikhney, A. G., Borges, C. R., Molodenskiy, D. S., Jeffries, C. M., and Svergun, D. I. (2020) SASBDB: Towards an automatically curated and validated repository for biological scattering data. *Protein Sci* **29**, 66-75
16. Trewhella, J., Duff, A. P., Durand, D., Gabel, F., Guss, J. M., Hendrickson, W. A., Hura, G. L., Jacques, D. A., Kirby, N. M., Kwan, A. H., Perez, J., Pollack, L., Ryan, T. M., Sali, A., Schneidman-Duhovny, D., Schwede, T., Svergun, D. I., Sugiyama, M., Tainer, J. A., Vachette, P., Westbrook, J., and Whitten, A. E. (2017) 2017 publication guidelines for structural modelling of small-angle scattering data from biomolecules in solution: an update. *Acta Crystallogr D* **73**, 710-728
